# Moving toward Personalized Medicine in the Methadone Maintenance Treatment Program: A Pilot Study on the Evaluation of Treatment Responses in Taiwan

**DOI:** 10.1155/2013/741403

**Published:** 2013-12-16

**Authors:** Hsin-Ya Lee, Jih-Heng Li, Yuh-Ling Sheu, Hsin-Pei Tang, Wei-Chiao Chang, Tze-Chun Tang, Yi-Chun Yeh, Shing-Yaw Wang, Ray-H. Liu

**Affiliations:** ^1^School of Pharmacy, College of Pharmacy, Kaohsiung Medical University, No. 100 Shih-Chuan 1st Road, Kaohsiung City 807, Taiwan; ^2^Program in Toxicology, College of Pharmacy, Kaohsiung Medical University, No. 100 Shih-Chuan 1st Road, Kaohsiung City 807, Taiwan; ^3^Department of Addiction and Forensic Psychiatry, Jianan Mental Hospital, No. 80, Lane 870, Jhong-Shan Road, Rende District, Tainan City 71742, Taiwan; ^4^School of Pharmacy, Taipei Medical University, No. 250 Wu-Shin Street, Taipei 110, Taiwan; ^5^Department of Psychiatry, Kaohsiung Medical University Hospital, No. 100 Shih-Chuan 1st Road, Kaohsiung City 807, Taiwan; ^6^Department of Psychiatry, Faculty of Medicine, College of Medicine, Kaohsiung Medical University, No. 100 Shih-Chuan 1st Road, Kaohsiung City 807, Taiwan; ^7^Department of Medical Technology, Fooyin University, No. 151 Ching-Hsueh Road, Kaohsiung City 831, Taiwan

## Abstract

This pilot study simultaneously evaluated the effects of various factors, including genetic variations of *CYP2B6*, *CYP2C19*, and *ABCB1*, demographic characteristics, disease states, methadone-drug interactions (MDIs), and poly-substance use, on the treatment responses among non-HIV patients in the methadone maintenance treatment program (MMTP) in Taiwan. A total of 178 patients were recruited from two major hospitals that provided MMTP services in southern Taiwan, and information regarding concomitant medications and diseases was acquired from the National Health Insurance (NHI) program. The results demonstrated that the methadone maintenance dose, *CYP2B6* 785G allele, and *ABCB1* 2677T allele have positive effects on the methadone plasma concentration. In contrast, patients with HCV coinfection, alcohol problems, and psychiatric diseases may have a negative response to treatment. Thus, a comprehensive evaluation of treatment responses in the MMTP should include not only genetic polymorphisms in methadone metabolism and transporter proteins, but also concomitant diseases, MDIs, and poly-substance use. The results also suggest that personalized medicine may be indispensable for a better outcome of the MMTP.

## 1. Introduction

Methadone maintenance treatment programs (MMTPs) have been shown to be effective in reducing illicit drug use and risks of human immunodeficiency virus (HIV) and hepatitis C (HCV) infection [1–3]. Achievement of an optimal methadone plasma concentration is crucial for a successful MMTP [4]. However, due to wide interindividual variations in methadone pharmacokinetics [5–7], some patients' methadone plasma concentrations may be too difficult to reach within a therapeutic range even though they receive higher dosages. Between 30% and 80% of patients are considered poor responders to MMTPs [8], and 98.6% of injecting drug users (IDUs) on a MMTP still continue to inject drugs [9].

Some factors are thought to influence the methadone plasma concentration and treatment responses. IDUs may have severe medical complications of substance use disorders, including psychiatric disease (e.g., depression, anxiety) [10–12], infectious diseases (HIV, HCV) [13], and pain disorders [14].

In addition, many treatment conditions can result in complications. For example, methadone-drug interactions (MDIs) could occur because MMTP clients have a high tendency towards taking concomitant medications (72%) [15]. Approximately 48% of patients have at least one MDI, and the most common MDI is benzodiazepines (38.1%), such as alprazolam and estazolam. Moreover, methadone interactions with some antiretroviral agents are also ubiquitous in HIV-MMTP clients [16, 17]. Some antiretroviral agents are well-documented as cytochrome P450 (CYP450) 3A4, 2B6, or 2D6 strong inducers (e.g., efavirenz) [18] or inhibitors. In a case report [19], interruption in the use of lopinavir-ritonavir triggered Torsade de Pointes (TdP) by increasing the methadone plasma concentration, because lopinavir-ritonavir may induce metabolic clearance of methadone involving CYP3A4, 2B6, and 2D6 enzymes.

Observational and *in vitro* studies have suggested that *CYP2B6 *[20–23], *2C19* [21, 22], and *ABCB1* [22, 24] genetic polymorphisms have important roles in gene codes for methadone-metabolizing enzymes and transporter proteins (p-glycoprotein, P-gp). CYP2B6 has been demonstrated to be an important contributor to S-methadone metabolism, and CYP2C19 preferentially metabolizes R-methadone [21, 25]. S-methadone has been found to contribute to higher levels of dissatisfaction and the risk of QT interval prolongation [26, 27]. R-methadone has been reported to be associated with clinical effects as a result of its stronger activation of *μ*-opioid receptors [28]. Methadone is a substrate of P-gp, encoded by the multidrug resistance1 (*ABCB1*) gene, which has the ability to influence the bioavailability of orally administered methadone in the gastrointestinal tract and hepatocytes and has an effect on mediating methadone transport through the blood-brain barrier (BBB) [29, 30].

Even though joint genetic effects of CYP and P-gp on a MMTP have been demonstrated in some pharmacogenetic studies [20, 31–34], few studies have simultaneously considered other important factors, such as disease states, MDIs, and poly-substance use, in assessing the methadone treatment responses, particularly in the ethnic Chinese populations. It is especially important that medical professionals understand the efficacy of and crucial factors related to MMTPs.

In Taiwan, to encounter the escalating IDUs and HIV infections through needle sharing, the first MMTPs were implemented in July 2006 [35, 36]. However, a thorough evaluation on the treatment responses of a MMTP has not yet been conducted. Therefore, the main purpose of this pilot study was to simultaneously evaluate the influence of genetic variations of *CYP2B6*,* CYP2C19*, and *ABCB1,* disease states, MDIs, and poly-substance use on the methadone steady-state trough plasma level and treatment responses in MMTP patients. In addition, as the methadone plasma dose and plasma concentration may be severely interfered by antiretroviral drugs, we recruited non-HIV patients to avoid the impact of antiretroviral agents on the MMTP and to evaluate the potential factors related to the methadone plasma concentration and treatment responses more precisely.

## 2. Materials and Methods

### 2.1. Study Patients

This study was conducted from February 2010 to December 2011 at Jianan Mental Hospital of the Department of Health, the first mental hospital to implement a MMTP, and Chung-Ho Memorial Hospital of Kaohsiung Medical University, a major medical center in southern Taiwan. One hundred and seventy-eight patients with no HIV infection (confirmed by medical records) aged ≥20 years and who were not pregnant were recruited. To ensure that patients' methadone plasma concentrations were at the steady-state condition and have stable methadone doses, patients who had participated in the MMTP ≥  1 month were recruited.

### 2.2. Ethics Statement

All information was kept strictly confidential and used for research proposes only. The study was approved by the Institutional Review Boards of Jianan Mental Hospital (Approval number 10-002) and Chung-Ho Memorial Hospital (Approval number KMUH-IRB-980429). Written informed consent was obtained from all participants who were given a detailed description of the study and had the chance to clarify any questions. Before agreeing to join the study, all participants were informed of the purpose of the study and the role and functions of their participation. Participants had the right to decline to take part in this research. They could also stop participating in the research at any time. Treatments of participants and nonparticipants at the hospital were not discriminated in any way.

### 2.3. Clinical Assessments

The participants were interviewed by a trained research staff to collect information on their sociodemographic characteristics, histories of substance use, and adverse drug reactions. Sociodemographic characteristics included age, sex, weight, educational level (years of education completed), and current marital status. Substance use referred to the use of cigarettes, alcohol, or betel quid, while drug use included illicit use of heroin and amphetamines.

Data on the methadone maintenance dose, admission date to the MMTP, and data regarding hepatitis, including hepatitis B (HBV) or HCV, as defined by a serological blood test with the presence of hepatitis B surface antigen (HBsAg) or detected antibodies to HCV, respectively, were obtained from medical records.

Information regarding concomitant medications and diseases during the period of the MMTP was acquired from the National Health Insurance (NHI) program, a national universal health insurance program with a coverage rate of over 99% for all citizens in Taiwan. General medical and pharmacy records were obtained for all patients. MDIs in the Taiwan MMTP [15] were categorized as follows:

1. decreased methadone metabolism: by agents that could inhibit CYP3A4, 2B6, and 2D6 enzymes, such as cimetidine, ketoconazole, erythromycin, clarithromycin, and metronidazole;
2. increased methadone metabolism: by agents that could induce CYP3A4, 2B6, and 2D6 enzymes, such as dexamethasone, carbamazepine, spironolactone, and rifampine;
3. antianxiety drugs: benzodiazepines (BZDs) and non-BZDs, such as zolpidem and zopiclone;
4. antipsychotic drugs: chlorpromazine, prochlorperazine, thioridazine, and haloperidol;
5. opioid-related drugs: morphine, tramadol, nalbuphine, propoxyphene, and bupernorphine;
6. antidepressive drugs: imipramine, fluoxetine, sertraline, amitriptyline, paroxetine, fluroxamine, and risperidone;
7. QT prolongation drugs: droperidol, levofloxacin, moxifloxacin, and ciprofloxacin.

### 2.4. Treatment Responses

Response to treatment is defined by nonconsumption of heroin. Determination of nonconsumption of heroin (responders) was first identified by the patients' self-reporting, followed by confirmation of the negative urine results for illicit opiates use, whereas consumption of drugs (nonresponders) was based on the self-reporting of patients and confirmed by the positive urine results for opiates. Urinalysis results were retrieved from medical records. In Taiwan, poppy seeds are not a traditional food and possession or consumption of poppy seeds is an illegal act. Therefore, false positive results for morphine due to consumption of poppy seeds can be excluded. The urine was thus defined as positive for illicit opiates use when the amount of morphine in the urine sample was equal to or greater than 300 ng/mL, as determined by gas chromatography-mass spectrometry (GC-MS).

### 2.5. Plasma Sample Analysis

Blood samples were withdrawn prior to next methadone administration (trough methadone plasma concentration). After blood samples (5 mL) were drawn, they were centrifuged and the plasma was stored at −70°C until quantification of the enantiomers of methadone (R-methadone and S-methadone). The concentrations of enantiomers of methadone in the plasma were determined by capillary electrophoresis (CE-UV) [37] after liquid-liquid extraction (LLE) of samples with ethyl acetate/heptane (4 : 1, v/v). A Beckman P/ACE MDQ system with a UV detector (214 nm) was used for the enantioselective determination of methadone and atenolol as an internal standard (I.S.). After LLE, CE was performed in an uncoated fused-silica capillary of 31.2 cm (effective length 20 cm) with a 50-*μ*m internal diameter. A constant voltage of 20 kV was applied and the cartridge temperature was maintained at 25°C. The running buffer was 80 mM phosphate buffer and 3.3 mM carboxymethyl-*β*-cyclodextrin (CM-*β*-CD) (pH 3). The relative standard deviation (RSD) and relative error (RE) were less than 5.3% and 7.7%, respectively, and the limit of quantification (LOQ) was found to be 10 ng/mL.

### 2.6. Genetic Polymorphism Analysis

Genomic DNA was extracted from venous blood samples using a FlexiGene DNA kit (Qiagen, Hilden, Germany) according to the manufacturer's instructions. For detection of *CYP2B6 *A785G, G516T, and C1459T, *CYP2C19 *G681A, G636A, and C3402T, and *ABCB1* C1236T, G2677A, and C3435T single nucleotide polymorphisms (SNPs), the polymerase chain reaction-restriction fragment length polymorphism (PCR-RFLP) assay was used.

PCR reactions were performed in a reaction volume of 50 *μ*L with 100 ng genomic DNA, SapphireAmp Fast PCR Master Mix (Takara, Otsu, Shiga, Japan), and 25 pmole of each primer. The protocol was as follows: 35 cycles, with an initial denaturation step at 95°C for 10 seconds, followed by annealing at primer-specific temperatures (56.4°C−62.4°C) for 10 seconds, 20 seconds of extension at 72°C, and cooling to 4°C for 5 minutes. PCR fragments were amplified using a PCR Thermal Cycler Dice TP600 (Takara, Otsu, Shiga, Japan). For the *CYP2B6 *A785G, G516T, and C1459T, *CYP2C19 *G681A, G636A and C3402T, and* ABCB1* C1236T, G2677A, and C3435T SNPs, the restriction enzymes, *Bsr* I, *Sty* I, *Bgl* II, *Msp* I, *BamH* I, *MnI* I, *Hae* III, *BseY* I, and *Mbo* I, were applied, respectively. DNA fragments were separated by electrophoresis in 2% agarose gel.

The SNPs were also confirmed using direct sequencing. Amplicons were purified using PCR Clean Up-M (Viogene, Taiwan). The PCR products were then directly sequenced using a BigDye Terminator Cycle Sequencing Ready Reaction Kit and analyzed on an ABI 3730 DNA sequencer (Applied Biosystems, CA, USA).

### 2.7. Statistical Analysis

Hardy-Weinberg equilibrium was tested for each SNP. R,S-methadone, R-methadone, and S-methadone plasma concentrations were divided by the methadone dose (in milligrams per day) and by the patient weight (in kilograms). Then, the values were natural-log transformed before analysis. The Levene test was applied to verify homogeneity of variance.

For the purpose of assessing the statistical differences between responders and nonresponders and between *CYP2B6*, *CYP2C19*, and *ABCB1* genotypes, analysis of variance (ANOVA), or the *t*-test was used for data presenting a homogenous distribution, or the Kruskal-Wallis nonparametric test or the Mann-Whitney *U* test was used for those that did not attain the estimated homogeneity and normality for continuous variables. The chi-square test or Fisher's exact test was used for categorical variables to account for the small sample size.

Stepwise multiple linear regression analysis was performed in order to explore the variables independently related to methadone plasma concentrations. However, because methadone plasma concentrations were skewed positively, the methadone plasma concentrations were natural-logarithmically transformed to achieve a normal distribution. The variables included were age, weight, sex, sociodemographics, methadone dose, current amphetamine use, methadone treatment duration, drugs related to methadone-drug interactions, and *CYP2B6*, *CYP2C19*, and *ABCB1* genetic variability.

Moreover, stepwise multiple logistic regression analysis was conducted to identify associations between potentially predictive variables (sociodemographics, tobacco use, betel nut use, alcohol use, current amphetamines use, methadone treatment duration, HBV, HCV, pain disorders, depression, anxiety, psychiatric disorders, methadone maintenance dose, *CYP2B6*, *CYP2C19*, and *ABCB1* genetic variability) and treatment responses. A variable was selected using mixed stepwise regression and included if its *P* value was ≤0.05 and excluded if its *P*-value was ≥0.05.

All analyses were completed using JMP (version 9.0, SAS Institute, Cary, USA). The statistical tests performed were two-tailed and a *P* value <0.05 denoted a statistically significant difference.

## 3. Results

The results were based on the data of 178 patients, including 156 males and 22 females. The mean age (±SD) of the patients was 39.5 ± 7.1 years (range, 25–59 years), and the mean weight was 68.8 ± 12.8 kg (range, 40–118 kg). Of the patients, 160 (89.9%) and 33 (18.5%) had HCV and HBV coinfection, respectively, and 128 (72.7%) patients had coadministration of other medications with methadone. The mean methadone daily maintenance dose was 50.8 ± 30.5 mg (range 5–250 mg/d).

A lack of available genotype data in two male subjects resulted from methodological problems. The mean R,S-methadone, R-methadone, and S-methadone trough levels were 172.9 ± 150.6 ng/mL (range 6.9–1368.0 ng/mL), 92.9 ± 79.8 ng/mL (range 6.9–800.7 ng/mL), and 81.9 ± 73.2 ng/mL (range 7.3–567.3 ng/mL), respectively.

### 3.1. Demographic Characteristics, Disease State, *CYP2B6*, *CYP2C19*, and *ABCB1* Genotypes

The demographic characteristics, concomitant diseases, substance use history, and MDIs of patients (split into responders and nonresponders) are shown in Table 1. Responders and nonresponders differed significantly with respect to gender, alcohol use, HCV coinfection, psychiatric disorders, and benzodiazepine use. Although the responders and nonresponders had similar methadone doses, R,S-methadone, R-methadone, and S-methadone concentrations (Table 1), there were statistically significant differences in the distributions of *ABCB1* G2677T and C3435T between the responders and nonresponders (Table 2). No differences were found in the distribution of *CYP2B6* and *CYP2C19* genotypes between responders and nonresponders. *CYP2B6*, *CYP2C19,* and *ABCB1* allele frequencies observed in MMTP Taiwanese were in Hardy-Weinberg equilibrium (*P* > 0.05).

### 3.2. Methadone Maintenance Dose, Plasma Concentrations, and *CYP2B6*, *CYP2C19*, and *ABCB1* Genotypes

We investigated the trough R,S-methadone, R-methadone, S-methadone concentrations and methadone dose by the genotypes of the *CYP2B6*, *CYP2C19,* and *ABCB1* genes (Table 3). For *CYP2B6* A785G, though the methadone dose did not differ significantly (*P* = 0.42), the R,S-methadone plasma concentration was significantly different among the allele groups (*P* = 0.03); in particular, that of G/G carriers (5.84 ± 0.77 ng·kg/mL·mg) was higher than that of A/A carriers (5.35 ± 0.81 ng·kg/mL·mg).

For *ABCB1* C1236T, the T homozygous carriers (47.2 ± 24.7 mg) showed a trend towards a lower maintenance methadone dose than the T heterozygous carriers (50.4 ± 28.3 mg) and noncarriers (67.9 ± 50.4 mg) (*P* = 0.02). For *ABCB1* G2677T, the R,S-methadone (G/G group 5.23 ± 0.86 ng·kg/mL·mg versus G/T group 5.24 ± 0.73 ng·kg/mL·mg versus T/T group 5.67 ± 0.86 ng·kg/mL·mg, *P *= 0.008), R-methadone (G/G group 4.12 ± 0.76 ng·kg/mL·mg versus G/T group 4.26 ± 0.62 ng·kg/mL·mg versus T/T group 4.58 ± 0.65 ng·kg/mL·mg, *P* = 0.004), and S-methadone (G/G group 4.00 ± 0.68 ng·kg/mL·mg versus G/T group 4.08 ± 0.67 ng·kg/mL·mg versus T/T group 4.41 ± 0.77 ng·kg/mL·mg, *P* = 0.01) plasma concentrations differed significantly between the different genotypes.

Moreover, we studied the association between the methadone dose and the methadone plasma levels with different genotypes in the responders and nonresponders. The results did not differ significantly in terms of *CYP2B6* and *CYP2C19* genetic variability, with the exception of the *ABCB1* G2677T genotype (Table 4). In the responders, the R,S-methadone (G/G group 5.20 ± 0.98 ng·kg/mL·mg versus G/T group 5.00 ± 0.72 ng·kg/mL·mg versus T/T group 5.97 ± 0.71 ng·kg/mL·mg, *P* = 0.001), R-methadone (G/G group 4.09 ± 0.83 ng·kg/mL·mg versus G/T group 4.15 ± 0.51 ng·kg/mL·mg versus T/T group 4.72 ± 0.72 ng·kg/mL·mg, *P* = 0.01), and S-methadone (G/G group 3.95 ± 0.76 ng·kg/mL·mg versus G/T group 3.93 ± 0.61 ng·kg/mL·mg versus T/T group 4.55 ± 0.75 ng·kg/mL·mg, *P* = 0.01) plasma concentrations were significantly different among the different genotypes, although the methadone dose did not differ significantly among the allele groups. This result suggested that *ABCB1* gene polymorphism may play an important role in the methadone plasma concentrations.

### 3.3. Factors Associated with Methadone Plasma Concentrations and Treatment Responses

Stepwise multiple linear regression analysis revealed that the methadone maintenance dose (*β* = 0.0006, *P* = 0.001),* CYP2B6* A785G (*β* = 0.44, *P* = 0.03), and *ABCB1* G2677T (*β* = 0.37, *P* = 0.003) were independent predictors determining the R,S-methadone plasma concentration (Table 5). These results showed that the methadone maintenance dose, *CYP2B6* A785G, and *ABCB1* G2677T were positively associated with the R,S-methadone plasma concentration.

Stepwise logistic regression analysis was performed to identify associations of variables with treatment responses. Adjusted analyses showed that significant correlates for nonresponders were HCV coinfection (adjusted odds ratio, AOR  =  6.42, *P*  =  0.03), psychiatric diseases (AOR  =  2.71, *P* = 0.02), alcohol problems (AOR  = 2.25, *P* = 0.02), and *CYP2B6* A785G (AOR  = 1.74, *P* = 0.10). However, *ABCB1* G2677T (AOR  = 0.48, *P* = 0.05) and the female gender (AOR = 0.44, *P* = 0.09) were associated with a reduced odds of positive urine test with morphine (Table 6).

## 4. Discussion

A successful methadone maintenance treatment program (MMTP) for opioid-dependent users is associated with the optimal methadone dosage and methadone plasma concentrations. A number of factors related to the optimal methadone dosage and methadone plasma concentrations, including poly-substance use, concomitant diseases, MDIs, genetic polymorphisms in metabolism enzymes, *CYP2B6* and *CYP2C19*, and transporter proteins, *ABCB1*, were investigated comprehensively in our study. We observed that the methadone maintenance dose, the *CYP2B6* 785G allele, and the *ABCB1* 2677T allele have positive effects on the methadone plasma concentrations. Furthermore, a protective factor associated with treatment response was the ABCB1 2677T allele and the CYP2B6 785G allele, and the risk factors were HCV infection, alcohol problems, and diagnosis with a psychiatric disease.

Many Taiwan IDUs had an experience of sharing needles or dilution water, and the HIV prevalence among IDUs reached a peak in 2005 [35, 36]. However, many antiretroviral agents [18, 19] have been investigated as CYP3A4, 2B6, or 2D6 strong inducers or inhibitors. In order to avoid an influence of antiretroviral agents on the pharmacokinetics of methadone, we recruited non-HIV patients in this study. Therefore, the data derived from this study can provide more precise evidence in terms of predicting the methadone plasma concentrations and treatment responses.

Previous studies regarding *ABCB1* pharmacogenetics indicated that individuals with the 3-locus genotype pattern TT-TT-TT (C1236T, G2677T, and C3435T) have an approximately 5-fold chance of requiring a higher methadone dose [38]. Patients with C3435T alleles were more likely to require a higher methadone dose than noncarriers [31]. However, the genetic effects of P-gp on the methadone plasma concentration and treatment responses remain unclear.

In this study, we found that the *ABCB1* 2677TT allele has positive effects on methadone plasma concentrations and treatment responses. Subjects with mutations in C1236T, G2677T, or C3435T may have a lower P-gp expression or function at the BBB, such that the CNS exposure to methadone is increased, and a lower dose is required to prevent overdoses [24]. It has been shown that a synonymous SNP in C1236T is linked to G2677T and C3435T SNPs [39]. Thus, we observed that the effects of *ABCB1* G2677T on methadone plasma concentrations were similar to those of C1236T in this study. 1236T homozygous carriers needed lower maintenance doses (47.2 ± 24.7 mg) than heterozygous carriers (50.4 ± 28.3 mg) and noncarriers (67.9 ± 50.4 mg). The effects of *ABCB1* genetic polymorphism on the methadone dose in this study were also consistent with the results of Coller et al. [24], who showed that TT-TT (G2677T, C3435T) carriers (38.0 ± 16.8 mg) required a lower methadone dose than noncarriers (61.3 ± 24.6 mg). However, conflicting studies on the effect of *ABCB1* genetic variability on methadone dose and plasma concentrations have been published [20, 31]. Crettol et al. [20] showed that 2677TT carriers (2.75 ng/mL·mg) had a lower R,S-methadone plasma level than 2677GT carriers (3.23 ng/mL·mg) and 2677GG carriers (3.46 ng/mL·mg). Their study was conducted in 5 methadone dispensing centers in Geneva, Bern, Montreux, Lausanne, and Switzerland. Hung et al. [31] revealed that 3435T carriers have 2.58-fold to require higher methadone dose than noncarriers.

The *CYP2B6* 785GG allele also has positive effects on methadone plasma concentrations. The effects of *CYP2B6* genetic polymorphism on the methadone dose and methadone plasma concentrations in this study were consistent with the results of Levran et al. [38], who showed that Israeli Jewish subjects with 785GG (596.7 ng/mL) had a higher R,S-methadone plasma level than those with 785AA (514.9 ng/mL), while 785GG carriers (88.3 mg) had a lower methadone dose than 785AA (151.4 mg). Our study revealed that patients with 785GG (5.84 ng·kg/mL·mg) had a higher R,S-methadone plasma level than those with 785AA (5.35 ng·kg/mL·mg), while 785GG carriers (40.9 mg) had a lower methadone dose than 785AA (51.4 mg). Fonseca et al. [34] reported that the contributions to clinical treatment responses from *ABCB1*, *CYP2B6*, and *CYP2D6* genetic polymorphism are marginal. Nevertheless, these controversial results may be explained by different ethnicities or characteristics of participants.

We observed that 89.9% (160/178) of the patients were infected with HCV, but only 3.4% (6/178) of the patients received HCV therapy (ribavirin plus peginterferon alpha 2A or 2B). A very low proportion of MMTP patients receive HCV treatment [40]. HCV treatment with pegylated interferon-alfa plus ribavirin is often complicated by psychiatric side effects in patients with drug addiction because depression, anxiety, fatigue, flu-like syndromes, and irritability are typical interferon-alfa-associated adverse events. Patients can have an increased risk of discontinuing HCV treatment early in the first three months when most psychiatric adverse events appear and flu-like syndromes may be misunderstood as withdrawal syndromes [41]. However, MMTP clients who do not accept HCV treatment may also have similar uncomfortable feelings, such as fatigue, nausea, loss of appetite, muscle ache, flu-like symptoms, and depression, which may be mistaken as withdrawal syndromes as well. Relapse of illicit drug abuse may then follow. Thus, patients with HCV coinfection may have an increased risk to positive urine tests for morphine.

Poly-substance use, such as consumption of amphetamines, betel nut, cigarettes, and alcohol, has been found to be common among Taiwan MMTP patients [15], especially alcohol drinking [42]. A high proportion of Taiwanese MMTP patients have alcohol problems (31.4%) [42]; this is also true in other countries (41–52%) [43]. Alcohol problems have a negative effect on illicit opioid use. Coconsumption of methadone with alcohol is not only associated with road traffic crashes [44] but also related to an increased risk of relapsing into illicit drug use and discharge from the MMTP, particularly in females [45]. Therefore, alcohol problems among MMTP patients should be monitored closely.

Psychiatric comorbidity, such as schizophrenia, low mood, anxiety, hallucinosis, and panic disorders, often coexists in MMTP patients (78%) [46, 47]. Patients with psychiatric comorbidity may require a higher maintenance dose (154 ± 84 mg) than patients (99 ± 49 mg) without psychiatric disorders [48]. About 35% of MMTP patients with concurrent psychiatric diseases were found to be regular or problem users of BZDs, and they were more likely to have opioid-positive urine screens during the MMTP [49]. In addition, concurrent psychiatric diseases may reduce quality of life in MMTP clients [47].

There was a tendency that the *CYP2B6* 785GG allele has negative effects on treatment responses (AOR  = 1.74, *P* = 0.10). Previous studies [21, 32, 33] found that *CYP2B6* enzyme genetic polymorphisms were related to S-methadone metabolism, which often contributed to uncomfortable feelings and dissatisfaction with the MMTP [27]. Additionally, CYP2B6 slow metabolizers exhibit a reduced ability to metabolize S-methadone and were associated with an increased risk of a prolonged QT interval (OR = 4.5) [26], which may increase the risk of cardiac arrhythmias and sudden death.

The mean methadone maintenance dose in this study was 50.8 ± 30.5 mg, and in other studies Taiwan or Chinese MMTP patients ranged from 35 mg to 54.7 mg [50, 51]. However, the mean methadone maintenance dose was found to vary from 59.2 mg to 134 mg in Caucasians [20, 24, 32, 34], which is much higher than that in ethnic Chinese. The interethnicity or interindividual differences may result from genetic polymorphisms in metabolism enzymes and transporter proteins. We also detected *CYP2B6* C1459T genetic variability in our study and found that all MMTP clients were heterozygous carriers. The frequency of the *CYP2B6* 1459T allele in these Taiwan MMTP patients was about 50%, while it was 11.2% in Caucasians [20, 33]. This may be explained by patients with C1459T mutations having a significantly reduced *CYP2B6* protein expression, which decreases the enzymatic activity [52]. Hence, patients in Taiwan MMTPs may have a lower CYP2B6 metabolism enzyme activity and consequently require a lower methadone maintenance dose than Caucasians. The relationship between *CYP2B6* C1459T genetic polymorphisms and methadone maintenance dose among different ethnicities may be worthy of investigation in the future.

These results should be interpreted within the context of the following limitations. This study was conducted in southern Taiwan, and the results may not be generalized to other regions in Taiwan. In this study, we only recruited non-HIV patients, but some patients may use psychoactive drugs, which may interfere with methadone metabolic disposition. We also observed that psychoactive drugs or other drugs may not be key factors affecting on methadone plasma concentrations (Table 5). Finally, because MMTP patients may have different attitudes towards or habits related to the treatment of diseases, some may use over-the-counter (OTC) medications or Chinese herbal medicines to alleviate withdrawal symptoms. Therefore, the interactions of methadone with OTC drugs or Chinese herbal medicines may be underestimated among MMTP patients who used additional OTC medications or Chinese herbal medicines, and we did not consider these effects in assessing the methadone plasma concentrations and treatment responses.

In summary, we controlled some important factors, such as antiretroviral agents, which may affect the pharmacokinetics of methadone severely and confounded (or obscured) related variables that may have an impact on the methadone plasma concentrations and treatment responses. Therefore, the variables explored in this study can provide more precise evidence in predicting the methadone plasma concentrations and treatment responses.

The results of our study demonstrated that the methadone maintenance dose, *CYP2B6* 785G allele, and *ABCB1* 2677T allele have positive effects on the methadone plasma concentrations. Furthermore, a positive indicator of treatment responses is the *ABCB1* 2677T allele. In contrast, patients with HCV coinfection, alcohol problems, and psychiatric diseases may have negative treatment responses. Thus, the results suggest that a comprehensive evaluation of treatment responses in the MMTP should include not only genetic polymorphisms in methadone metabolism and transporter proteins, but also concomitant diseases, MDIs, and poly-substance use. This pilot study also provides clues that personalized medicine may play an important role in determining a better outcome of the MMTP.

## Figures and Tables

**Table 1:** Patient characteristics of responders and nonresponders to the Taiwan methadone maintenance treatment program (MMTP) based on urine morphine screen tests (*n* = 178).

Variables	Responders (*n* = 62)	Nonresponders (*n* = 116)	*P* value
Age, yr (SD)	39.1 (7.3)	39.6 (7.0)	0.67
Weight, kg (SD)	71.0 (14.5)	68.0 (12.1)	0.14
Gender, *n* (%)			
Male	50 (32.1)	106 (67.9)	0.03*
Education, *n* (%)			
Below high school	39 (36.4)	68 (63.6)	0.57
High school or above	23 (32.4)	48 (67.6)	
Marital status, *n* (%)			
Married or living with partner	14 (31.1)	31 (68.9)	0.65
Never married	36 (37.5)	60 (62.5)	
Divorced/widowed	11 (30.6)	25 (69.4)	
Dose, mg (SD)	51.6 (30.8)	50.4 (30.5)	0.78
R,S-Methadone, ng/mL (SD)	172.9 (134.9)	172.9 (158.8)	0.99
R-Methadone, ng/mL (SD)	92.9 (66.5)	92.9 (86.3)	0.99
S-Methadone, ng/mL (SD)	81.2 (70.6)	82.3 (74.8)	0.92
Substance use history, *n* (%)			
Tobacco	55 (36.7)	95 (63.3)	0.23
Alcohol	26 (44.8)	32 (55.2)	0.03*
Betel nut	21 (43.8)	27 (56.3)	0.15
Current amphetamine use, *n* (%)	10 (27.1)	27 (72.9)	0.52
Treatment duration, mo (SD)	19.4 (13.9)	17.4 (13.9)	0.37
Heroin use history, yr (SD)	7.8 (5.7)	8.5 (5.6)	0.48
HBV, *n* (%)	13 (39.4)	20 (60.6)	0.55
HCV, *n* (%)	60 (37.5)	100 (62.5)	0.03*
Pain disorders, *n* (%)	25 (39.1)	39 (60.9)	0.37
Depression, *n* (%)	11 (44.0)	14 (56.0)	0.29
Anxiety, *n* (%)	15 (46.9)	17 (53.1)	0.11
Psychiatric disorders^a^, *n* (%)	15 (57.6)	11 (42.3)	0.01*
Methadone-drug interaction			
(1) Increase methadone metabolism, *n* (%)	17 (46.0)	20 (54.0)	0.11
(2) Decrease methadone metabolism, *n* (%)	9 (36.0)	16 (64.0)	0.89
(3) Benzodiazepines (BZD) or non-BZD, *n* (%)	18 (56.2)	14 (43.8)	0.007*
(4) Antipsychotic drugs, *n* (%)	2 (28.6)	5 (71.4)	0.72
(5) Opioid-related drugs, *n* (%)	12 (40.0)	18 (60.0)	0.53
(6) Antidepressive drugs, *n* (%)	5 (50.0)	5 (50.0)	0.32
(7) QT prolongation, *n* (%)	7 (35.0)	13 (65.0)	0.98

^a^Psychiatric disorders included schizophrenic disorders, hallucinosis, paranoia, panic disorders, and neurotic disorders.

*Statistical significance set at *P* < 0.05; comparisons were performed by the Mann-Whitney *U* test, *t*-test, Chi-square test, or Fisher's exact test as appropriate.

**Table 2:** Frequencies of *CYP2B6*, *CYP2C19,* and *ABCB1* polymorphisms in responders and nonresponders to treatment (*n* = 176)^a^.

Genotype	Responders (*n* = 61)	Nonresponders (*n* = 115)	*P* value
*CYP2B6 *			
A785G (*4)			
A/A	29 (47.5)	72 (62.6)	0.15
A/G	26 (42.6)	35 (30.4)	
G/G	6 (9.9)	8 (7.0)	
G516T (*9)			
G/G	38 (62.3)	86 (74.8)	0.10
G/T	19 (31.1)	27 (23.5)	
T/T	4 (6.6)	2 (1.7)	

*CYP2C19 *			
G651A (*2)			
G/G	27 (44.3)	63 (54.8)	0.21
G/A	32 (52.5)	45 (39.1)	
A/A	2 (3.2)	7 (6.1)	
G636A (*3)			
G/G	53 (86.9)	105 (91.3)	0.14
G/A	6 (9.8)	10 (8.7)	
A/A	2 (3.3)	0 (0)	
C3402T (*17)			
C/C	60 (98.4)	114 (99.1)	0.64
C/T	1 (1.6)	1 (0.9)	
T/T	0 (0)	0 (0)	

*ABCB1* Genotype			
C1236T			
C/C	5 (8.2)	15 (13.0)	0.53
C/T	30 (49.2)	49 (42.6)	
T/T	26 (42.6)	51 (44.4)	
G2677T			
G/G	22 (36.1)	26 (22.6)	0.03*
G/T	21 (34.4)	63 (54.8)	
T/T	18 (29.5)	26 (22.6)	
C3435T			
C/C	30 (49.2)	39 (33.9)	0.02*
C/T	20 (32.8)	63 (54.8)	
T/T	11 (18.0)	13 (11.3)	

^a^No available data on genotype for two subjects (1 responder and 1 nonresponder) due to methodological problems.

*Statistical significance set at *P* < 0.05; comparisons were performed by the Chi-square test or Fisher's exact test as appropriate.

**Table 3:** Influence of *CYP2B6*, *CYP2C19,* and *ABCB1* polymorphism on methadone plasma concentrations (*n* = 176)^a^.

Gene	*n* (%)	Dose (SD)	R,S-Methadone^b^ (SD)	R-Methadone^b^ (SD)	S-Methadone^b^ (SD)
*CYP2B6* genotype					
A785G (*4)					
A/A	101 (57.4)	51.4 (26.5)	5.35 (0.81)	4.32 (0.69)	4.15 (0.67)
A/G	61 (34.6)	52.6 (38.2)	5.22 (0.81)	4.21 (0.66)	4.05 (0.72)
G/G	14 ( 8.0)	40.9 (18.4)	5.84 (0.77)	4.60 (0.79)	4.52 (0.89)
*P* value	—	0.42	0.03*	0.15	0.08
G516T (*9)					
G/G	124 (70.5)	50.4 (24.9)	5.28 (0.82)	4.29 (0.67)	4.10 (0.67)
G/T	46 (26.1)	54.4 (43.1)	5.42 (0.76)	4.29 (0.70)	4.21 (0.78)
T/T	6 (3.4)	36.7 (19.7)	5.99 (0.99)	4.57 (0.99)	4.57 (1.02)
*P* value	—	0.38	0.09	0.63	0.22

*CYP2C19* genotype					
G651A (*2)					
G/G	90 (51.1)	52.6 (35.2)	5.40 (0.79)	4.33 (0.74)	4.18 (0.78)
G/A	77 (43.8)	49.8 (24.9)	5.28 (0.86)	4.29 (0.62)	4.12 (0.64)
A/A	9 (5.1)	45.2 (26.8)	5.38 (0.68)	4.24 (0.72)	3.95 (0.62)
*P *value	—	0.71	0.66	0.90	0.60
G636A (*3)					
G/G	158 (89.8)	52.1 (31.3)	5.33 (0.83)	4.30 (0.69)	4.15 (0.70)
G/A	16 (9.1)	39.1 (20.9)	5.48 (0.70)	4.28 (0.64)	4.08 (0.87)
A/A	2 (1.1)	62.5 (31.8)	5.81 (0.13)	4.85 (0.16)	4.40 (0.66)
*P* value	—	0.24	0.55	0.52	0.81
C3402T (*17)					
C/C	174 (98.9)	49.9 (26.7)	5.35 (0.82)	4.30 (0.69)	4.15 (0.72)
C/T	2 (1.1)	140 (155.6)	4.55 (0.32)	4.18 (0.53)	3.79 (0.91)
T/T	0 (0)	0 (0)	0 (0)	0 (0)	0 (0)
*P* value	—	<0.0001*	0.08	0.81	0.47

*ABCB1* genotype					
C1236T					
C/C	19 (10.8)	67.9 (50.4)	5.23 (0.66)	4.36 (0.52)	4.21 (0.48)
C/T	80 (45.5)	50.4 (28.3)	5.38 (0.82)	4.29 (0.73)	4.17 (0.72)
T/T	77 (43.7)	47.2 (24.7)	5.37 (0.85)	4.29 (0.68)	4.10 (0.76)
*P* value	—	0.02*	0.75	0.91	0.79
G2677T					
G/G	48 (27.3)	49.6 (39.4)	5.23 (0.86)	4.12 (0.76)	4.00 (0.68)
G/T	84 (47.7)	53.1 (27.3)	5.24 (0.73)	4.26 (0.62)	4.08 (0.67)
T/T	44 (25.0)	48.5 (25.5)	5.67 (0.86)	4.58 (0.65)	4.41 (0.77)
*P* value	—	0.68	0.008*	0.004*	0.01*
C3435T					
C/C	69 (39.2)	56.8 (39.3)	5.26 (0.87)	4.31 (0.69)	4.13 (0.63)
C/T	83 (47.2)	47.1 (21.6)	5.36 (0.74)	4.26 (0.71)	4.12 (0.76)
T/T	24 (13.6)	47.7 (26.9)	5.52 (0.91)	4.49 (0.58)	4.27 (0.78)
*P* value	—	0.13	0.41	0.36	0.63

^a^No available data on genotype for two subjects due to methodological problems.

^b^R,S-Methadone, R-methadone, and S-methadone plasma concentrations were divided by the methadone dose (in milligrams per day) and by the patient weight (in kilograms). The unit of concentration is ng·kg/mL·mg. Then, the values were natural log transformed before analysis. All values are expressed as the mean and standard deviation (SD).

*Statistical significance set at *P* < 0.05; comparisons were performed by the *t*-test, ANOVA, Mann-Whitney *U* test, or Kruskal-Wallis test as appropriate.

**Table 4:** Influence of *ABCB1* polymorphism on methadone plasma concentrations in responders and nonresponders (*n* = 176)^a^.

*ABCB1* genotype	*n* (%)	Dose (SD)	R,S-Methadone^b ^(SD)	R-Methadone^b ^(SD)	S-Methadone^b ^(SD)
Responders (*n* = 61)					
G2677T					
G/G	22 (36.1)	47.6 (31.4)	5.20 (0.98)	4.09 (0.83)	3.95 (0.76)
G/T	21 (34.4)	61.3 (29.8)	5.00 (0.72)	4.15 (0.51)	3.93 (0.61)
T/T	18 (29.5)	47.1 (30.1)	5.97 (0.71)	4.72 (0.72)	4.55 (0.75)
*P* value	—	0.24	0.001*	0.01*	0.01*

^a^No available data on genotype for two subjects due to methodological problems.

^b^R,S-Methadone, R-methadone, and S-methadone plasma concentrations were divided by the methadone dose (in milligrams per day) and by the patient weight (in kilograms). The unit of concentration is ng·kg/mL·mg. Then, the values were natural log transformed before analysis. All values are expressed as the mean and standard deviation (SD).

*Statistical significance set at *P* < 0.05; comparisons were performed by the ANOVA or Kruskal-Wallis test as appropriate.

**Table 5:** Methadone maintenance dose and *CYP2B6* and *ABCB1* gene mutation relationships with R,S-methadone plasma concentration^a, b^.

Variable	Regression coefficient (*β*)	95% CI	*P* value
Dose (mg)	0.0006	0.002–0.009	0.001*
*CYP2B6* A785G			
A/G, G/G (versus A/A)	0.44	0.049–0.839	0.03*
*ABCB1* G2677T			
G/T, T/T (versus G/G)	0.37	0.124–0.616	0.003*

^a^R,S-Methadone concentration was natural-logarithmically transformed to achieve a normal distribution.

^b^Results are from stepwise multiple linear regression analysis. Age, marital status, weight, current amphetamine use, treatment duration, drugs related to methadone-drug interactions*, CYP2B6* G516T (*9), *CYP2C19* G681A/C (*2), G636A (*3), C3402T (*17), *ABCB1* C1236T, and C3435T were not significantly associated. Only variables significantly contributing to the models are displayed (variables selected using mixed stepwise regression); *P* value for model: <0.0001.

**Table 6:** Variables significantly associated with nonresponders in the Taiwan methadone maintenance treatment program (MMTP)^a^.

Variable	AOR^c^	95 % CI^d^	*P* value
Sex			
Female (versus male)	0.44	0.86–6.24	0.09
*CYP2B6* A785G (*4)			
A/G, G/G (versus A/A)	1.74	0.89–3.46	0.10
*ABCB1* G2677T/A			
G/T, T/T (versus G/G)	0.48	0.23–1.01	0.05
Psychiatric disorders^b^	2.71	1.10–6.53	0.02*
Alcohol use history	2.25	1.12–4.59	0.02*
HCV	6.42	1.14–121.3	0.03*

^a^Age, marital status, tobacco use, betel nut use, current amphetamine use, treatment duration, HBV, pain disorders, depression, anxiety, methadone maintenance dose, *CYP2B6* G516T, *CYP2C19* G681A/C, G636A, C3402T,* ABCB1 *C1236T, and C3435T were not significantly associated. In the multiple logistic regression model, variables were selected using mixed stepwise regression; *P* value for model: 0.0002.

^b^Psychiatric disorders included schizophrenic disorders, hallucinosis, paranoia, panic disorders, and neurotic disorders.

^c^AOR, adjusted odds ratio.

^d^95% CI, 95% confidence interval.
